# Evaluation of Xpert MTB/RIF and MODS assay for the diagnosis of pediatric tuberculosis

**DOI:** 10.1186/1471-2334-13-31

**Published:** 2013-01-23

**Authors:** Nguyen Thi Quynh Nhu, Dang Thi Minh Ha, Nguyen Duc Anh, Do Dang Anh Thu, Tran Ngoc Duong, Nguyen Dang Quang, Nguyen Thi Ngoc Lan, Tran Van Quyet, Nguyen Thi Bich Tuyen, Vo Thi Ha, Do Chau Giang, Nguyen Huy Dung, Marcel Wolbers, Jeremy Farrar, Maxine Caws

**Affiliations:** 1 Oxford University Clinical Research Unit, Hospital for Tropical Diseases, 764 Vo Van Kiet, District 5, Ho Chi Minh City, Vietnam; 2 Pham Ngoc Thach Hospital for Tuberculosis and Lung Diseases, 120 Hung Vuong, District 5, Ho Chi Minh City, Vietnam; 3 Centre for Clinical Vaccinology and Tropical Medicine, Churchill Hospital, Old Road, Headington, Oxford, UK

**Keywords:** Tuberculosis, GeneXpert MTB/RIF, MODS, Pediatric, Childhood

## Abstract

**Background:**

Tuberculosis (TB) in children is rarely confirmed due to the lack of effective diagnostic tools; only 10 to 15% of pediatric TB is smear positive due to paucibacillary samples and the difficulty of obtaining high-quality specimens from children. We evaluate here the accuracy of Xpert MTB/RIF in comparison with the Micoroscopic observation drug susceptibility (MODS) assay for diagnosis of TB in children using samples stored during a previously reported evaluation of the MODS assay.

**Methods:**

Ninety-six eligible children presenting with suspected TB were recruited consecutively at Pham Ngoc Thach Hospital in Ho Chi Minh City Viet Nam between May to December 2008 and tested by Ziehl-Neelsen smear, MODS and Mycobacterial growth Indicator (MGIT, Becton Dickinson) culture. All samples sent by the treating clinician for testing were included in the analysis. An aliquot of processed sample deposit was stored at −20°C and tested in the present study by Xpert MTB/RIF test. 183 samples from 73 children were available for analysis by Xpert. Accuracy measures of MODS and Xpert were summarized.

**Results:**

The sensitivity (%) in detecting children with a clinical diagnosis of TB for smear, MODS and Xpert were 37.9 [95% CI 25.5; 51.6], 51.7 [38.2; 65.0] and 50.0 [36.6; 63.4], respectively (per patient analysis). Xpert was significantly more sensitive than smear (P=0.046). Testing of additional samples did not increase case detection for MODS while testing of a second sputum sample by Xpert detected only two additional cases. The positive and negative predictive values (%) of Xpert were 100.0 [88.0; 100.0] and 34.1 [20.5; 49.9], respectively, while those of MODS were 96.8 [83.3; 99.9] and 33.3 [19.6; 49.5].

**Conclusion:**

MODS culture and Xpert MTB/RIF test have similar sensitivities for the detection of pediatric TB. Xpert MTB RIF is able to detect tuberculosis and rifampicin resistance within two hours. MODS allows isolation of cultures for further drug susceptibility testing but requires approximately one week to become positive. Testing of multiple samples by xpert detected only two additional cases and the benefits must be considered against costs in each setting. Further research is required to evaluate the optimal integration of Xpert into pediatric testing algorithms.

## Background

The World Health Organisation has estimated that there are 1 million cases of tuberculosis in children every year [1]. Seventy-five percent of these cases occur in the 22 high TB burden countries [2]. TB in children is rarely confirmed due to the lack of effective diagnostic tools; only 10 to 15% of pediatric TB is smear positive due to paucibacillary samples and the difficulty of obtaining high-quality specimens from children [3]. HIV co-infection further complicates diagnosis [4]. The overall focus of National TB programmes on notification of smear positive tuberculosis under the DOTS strategy has thus led to a ‘chronic neglect’ of pediatric TB, under the assumption that an overall decline in adult tuberculosis would also address pediatric TB [5,6]. Current best estimates of the burden of childhood TB are based upon multiple assumptions and the true burden of pediatric TB is unknown.

Diagnosis of pediatric TB is principally based on the evaluation of medical history, clinical evaluation with chest radiography, tuberculin skin-testing and smear microscopy with mycobacterial culture where available [7,8]. Diagnostic tests currently used for adult tuberculosis show low sensitivity when applied to children. Until the advent of the Xpert MTB/RIF test, nucleic acid amplification tests (NAAT) required a specialized laboratory with rigorous quality control which can be difficult to maintain in low resource settings. In the last decade, significant advances have been made in the development and implementation of novel diagnostic tests for TB in adults [9]. However, evaluation of the applicability of these tests for pediatric TB has not been extensive [6].

The non-commercial rapid liquid culture technique, microscopic observation drug susceptibility (MODS) assay [10] has a sensitivity for detection of TB in children similar to Mycobacterial growth indicator (MGIT) culture with faster turn-around times and can therefore significantly increase confirmation of TB in children compared to smear microscopy alone [11,12]. However, the plate reading is relatively labour intensive and cultures require approximately a week to become positive.

The Xpert MTB/RIF test (Cepheid, USA) has shown sensitivity and specificity approaching that of culture in adult sputum samples, 90.4% sensitivity and 98.4% specificity in a recent meta-analysis and has been endorsed by the WHO for use on sputum samples [13,14].

Scale-up implementation projects are underway in a number of countries, including Vietnam [15]. A recently published study of 452 children with suspected TB in South Africa, a high HIV incidence region, reported a doubling of the case detection with Xpert MTB/RIF compared to fluorescence smear, detecting 78% and 38%, respectively [16]. The test is a promising method to improve the diagnosis of pediatric TB, but limited data is available on the performance of the test with pediatric samples and is urgently required to determine appropriate applications for Xpert testing.

Vietnam is a high TB burden country with an estimated incidence of 200/100 000 population and an estimated 2.7% prevalence of MDR TB among new cases [17]. Twenty five percent of the Vietnamese population is under 15 years old [18]. In 2007, only 14.9% (59/395) of treated pediatric TB cases had microbiological confirmation at Pham Ngoc Thach hospital, the tertiary referral hospital for TB in southern Vietnam [11].

We evaluate here the accuracy of Xpert in comparison with the MODS assay for diagnosis of TB in children using samples stored during a previously reported evaluation of the MODS assay [11].

## Methods

### Recruitment

This study used stored samples and data from a previously reported prospective evaluation of the MODS assay for diagnosis of pediatric TB [11]. The protocol was approved by the Institutional Review Board (IRB) at Pham Ngoc Thach Hospital and the Health Services of Ho Chi Minh City. Informed consent was not sought because the study was conducted on routine samples only and it did not involve any intervention, additional samples or change in patient management. This patient consent waiver was approved by the IRB of Pham Ngoc Thach Hospital in the protocol.

All eligible children <16 years of age with clinical suspicion of tuberculosis presenting to the pediatric ward at Pham Ngoc Thach Hospital, Ho Chi Minh City, Viet Nam from May 2008 to December 2008 were included in the study [11]. Any patient already receiving TB therapy for more than seven days was excluded from the study**.**

Children were classified into 3 diagnostic categories, confirmed TB, probable TB or TB unlikely [11]. Children with confirmed TB had acid-fast bacilli observed by smear microscopy or *M.tuberculosis* isolated from a clinical sample by MGIT culture. Children with ‘probable TB’ had clinical symptoms consistent with TB, did not receive any alternative diagnosis and received TB treatment. Children in the ‘TB unlikely’ category recovered without TB treatment or received an alternative confirmed diagnosis.

The treating clinician determined the number and type of samples collected, following routine practice. No additional samples to routine care were collected as part of this study.

### Sample processing

During the original evaluation of the MODS assay, an aliquot of decontaminated sample deposit was stored at −20°C. These stored aliquots were used for current evaluation of Xpert MTB/RIF. In total, 183 samples from 73 patients were available for analysis, including sputum (n=126), gastric fluid (n=49), cerebral spinal fluid (CSF) (n=5), and pleural fluid (n=3). All of these samples, except CSF, were processed by standard decontamination using Sputaprep (NaOH –NALC 2%, Nam Khoa Company-Viet Nam) before storage.

Smear, MGIT and MODS were performed as described previously in accordance with standard protocols.

Ziehl-Neelsen (ZN) smear: Two drops (approximately 200 μl) of processed sample deposit were placed on a slide and stained by ZN method according to the WHO standard protocol [19].

MGIT: Five hundred microlitres of each deposit was inoculated into a supplemented MGIT tube following the manufacturer’s protocol. The culture was incubated in Bactec MGIT 960 system at 37°C and the result automatically reported.

MODS assay: MODS culture was prepared in a 48-well-tissue-culture plate. Two hundred and fifty microlitres of pellet was added to 7H9 medium supplemented with OADC and PANTA antibiotic solution. After at least 4 days of incubation, the growth of *M. tuberculosis* was recorded by reading through an inverse microscope.

Xpert MTB/RIF: The test was conducted on decontaminated sample deposits following the manufacturer’s standard operating procedure. The sample volume stored varied from 0.5 ml-1.5mls. Processed sample deposits exceeding 0.5 ml were centrifuged at 3,000 g for 15 minutes and excess supernatant discarded to obtain a final volume of 0.5 ml. One and a half milliliters of sample reagent was added to 0.5 ml of processed sample, and incubated at room temperature for 15 minutes with intermittent shaking and finally added to the test cartridge and loaded onto the machine. The result was available after 2 hours.

For any sample with a positive result for rifampicin resistance by Xpert MTB/RIF, the corresponding isolate from the same sample isolated by MGIT or MODS culture was tested for RIF and isoniazid (INH) susceptibility by MTBDR*plus* test (Hain Lifesciences, Germany) and Bactec Mycobacterial growth indicator tube SIRE Drug susceptibility test (MGIT SIRE DST, Becton Dickinson, USA) following the manufacturer’s protocol [20].

### Statistical analysis

Accuracy measures (sensitivity, specificity, positive and negative predictive values) of the evaluated tests for *M.tuberculosis* detection were determined using two gold standards for the reference diagnosis. The first comparison was made using microbiologically confirmed TB (by smear or MGIT) as the gold standard. The second comparison was made using a clinical diagnosis of TB (i.e. confirmed or probable TB) as the gold standard. Accuracy measures and corresponding confidence intervals were reported both on the patient and on the sample level.

For the per patient analyses, a patient was considered to be positive if the test showed a positive result for at least one of the patient’s samples. Exact Pearson-Clopper confidence intervals for accuracy measures are reported. Comparisons between the sensitivities of different diagnostic tests are based on McNemar’s test with continuity correction.

For the per sample analyses, confidence intervals for accuracy measures and comparisons of sensitivities of different tests are based on marginal logistic regression with an identity link function. Of note, using marginal models and generalized estimating equations accounts for potential dependence between test results of samples from the same patient. To account for the fact that specificities and positive predictive values were close to 100% leading to a separation problem for the marginal logistic regression, we used an Agresti-Caffo-type adjustment for the confidence intervals in these cases [21]. We also calculated time-dependent sensitivity curves for MGIT and MODS. For these, a test result was considered as positive by time t if the respective test was positive overall and reached the positive value at most t days after sample collection. Time-dependent sensitivity curves were estimated with the Kaplan-Meier method and samples without a positive test result were formally regarded as censored on day “infinity”.

The association between Ct values for probe A and smear grade was summarized and tested based on Spearman’s rank correlation.

All analyses were done using the statistical software R version 2.14.0 (R Foundation for statistical computing, Vienna, Austria). All reported confidence intervals are two-sided 95% confidence intervals and p-values ≤ 0.05 were considered as statistically significant.

## Results

**Figure 1:**
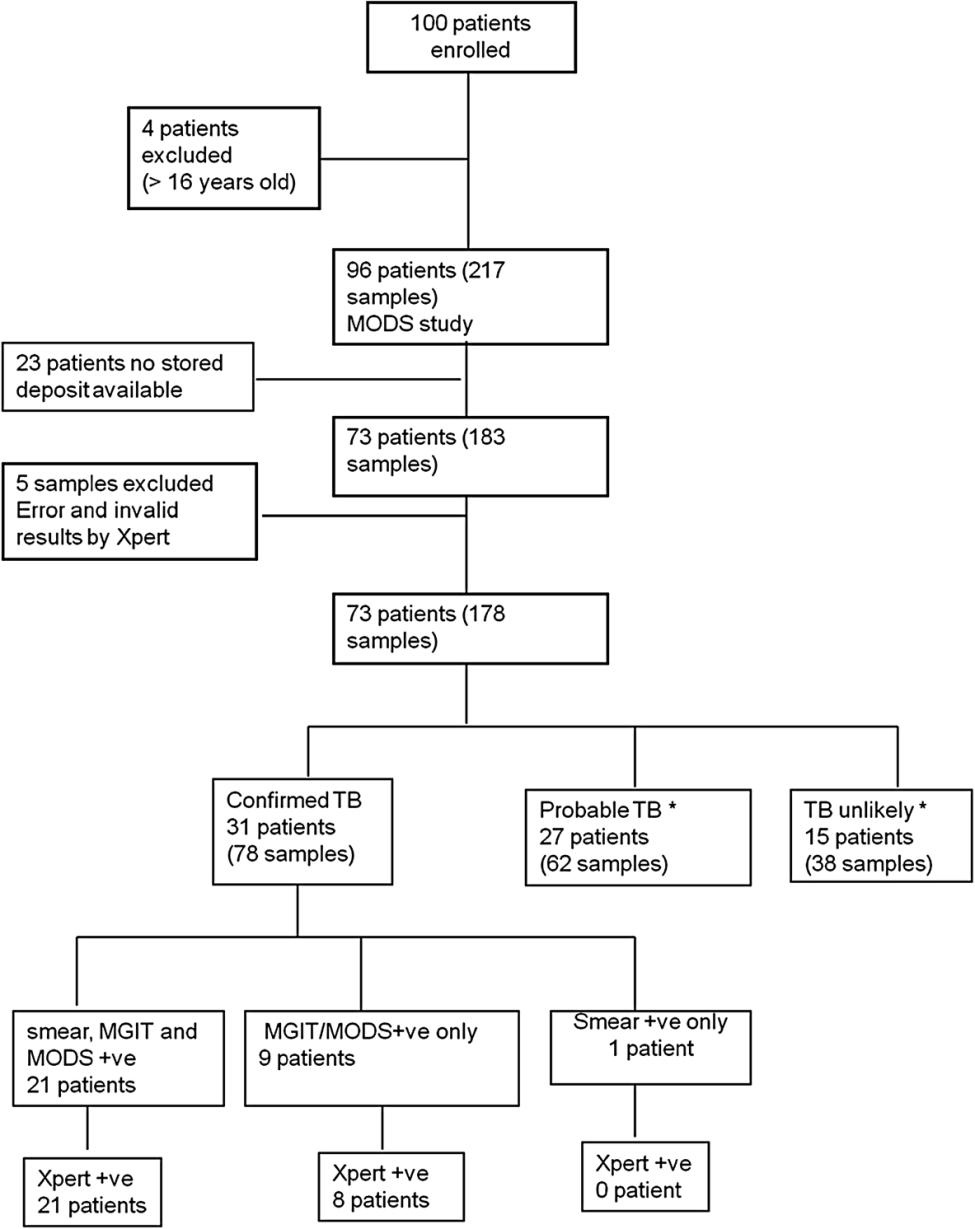
**Flow-chart of patient enrolment and analysis.** *All patients in ‘probable TB’ or ‘TB unlikely’ categories were Xpert negative.

The previously reported prospective evaluation of the MODS assay for diagnosis of pediatric TB [11] included data from 96 children but 23 children did not have a stored aliquot available for analysis by Xpert in the present study due to insufficient volume for storage or the use of the stored deposit aliquot for reculture in the original MODS study. The majority (69.6%, n=16/23) of these were in the probable TB category. Thus, data from 73 children were included in the present analysis (Figure 1). Almost half of these children (n=35/73, 47.9%) were between 11 and 16 years old, with 20.5% (n=15/73) 6–10 years old and 31.5% (n=23/73) 0–5 years of age.

There were 31/73 children (42.5%) with confirmed TB, 27/73 (37.0%) with probable TB and 15/73 (20.5%) were TB unlikely. The treating clinician determined the number and type of samples collected. For children aged five years or lower, the most common sample type was gastric aspirate, while for older children, sputum was normally collected. Only four children had two different sample types (Table 1).

**Table 1:** Sample types included by age of children

**Age (years old) by patients**	**Number of children provided sputum**	**Number of children provided gastric fluid**	**Number of children provided CSF**	**Number of children provided pleural fluid**	**Total**
0 – 5	5	18	2	0	28
6 – 10	11	0	3	1	15
11 – 16	33	2	0	2	34
Total	49	20	5	3	77*

* 4 patients provided 2 sample types.

HIV testing was not performed as part of the study, and only eight children had an HIV test as part of routine clinical care, in accordance with normal practice at PNT. Seven of eight children tested were HIV infected.

Amongst 183 samples from 73 children tested, there were 2 (1.1%) Xpert tests with invalid reports and 3 (1.6%) with error reports (‘post-run analysis’ for 2 tests, ‘operation terminated’ for 1 test). Error and invalid results were excluded from further analysis, resulting in a total of 178 samples but this did not decrease the number of patients (n=73).

### Accuracy of Xpert MTB/RIF

#### Per patient analysis (n=73)

The majority of patients (86%, 63/73) submitted multiple samples; 60% (44/73) of patients produced 3 samples and 26% (19/73) produced 2 samples.

In the ‘per patient’ analysis, Xpert showed a very similar sensitivity to MODS for both confirmed TB alone and all TB cases (confirmed + probable), 93.5% vs. 96.8% and 50.0% vs. 51.7%, respectively. Xpert was significantly more sensitive than smear (p= 0.046, same p-value for both gold standards) (Table 2). Relative to smear, seven additional cases were detected overall by Xpert while MODS detected eight additional cases over smear. Therefore, only a single case was detected by MODS which was not detected by Xpert.

**Table 2:** Sensitivity, specificity, positive and negative predictive values of smear, MODS, MGIT and Xpert (in%) for the diagnosis of pediatric tuberculosis

		**Sensitivity% (x/n); [95%CI]**	**Specificity% (x/n); [95%CI]**	**PPV% (x/n); [95%CI]**	**NPV% (x/n); [95%CI]**
Gold standard 1 = Microbiological confirmation	**Per patient analysis (n=73)**				
	Smear	71.0 (22/31) [52.0; 85.8])	N/a	N/a	82.3 (42/51) [69.1; 91.6]
	MGIT	96.8 (30/31) [83.3; 99.9]	N/a	N/a	97.7 (42/43) [87.7; 99.9]
	MODS	96.8 (30/31) [83.3; 99.9]	97.6 (41/42) [87.4; 99.9]	96.7 (30/31) [83.3; 99.9]	97.6 (41/42) [87.4; 99.9]
	Xpert	93.5 (29/31) [78.6; 99.2]	100 (42/42) [91.6; 100.0]	100.0 (29/29) [88.1; 100.0]	95.5 (42/44) [84.5; 99.4]
	**Per sample analysis (n=178)**				
	Smear	53.8 (42/78) [39.8; 67.9])	N/a	N/a	73.5 (100/136) [63.1; 83.9]
	MGIT	92.3 (72/78) [87.4; 97.2]	N/a	N/a	94.3 (100/106) [90.3; 98.4]
	MODS	84.6 (66/78) [78.1; 91.1]	99 (99/100) [94.3; 99.9]	98.5 (66/67) [91.6; 99.9]	89.2 (99/111) [83.3; 95.0]
	Xpert	79.5 (62/78) [70.6; 88.4])	100 (100/100) [95.7; 100.0]	100 (62/62) [93.3; 100.0]	86.2 (100/116) [78.9; 93.5]
Gold standard 2 = Clinical diagnosis	**Per patient analysis (n=73)**				
	Smear	37.9 (22/58) [25.5; 51.6]	N/a	N/a	29.4 (15/51) [17.5; 43.8]
	MGIT	51.7 (30/58) [38.2 ; 65.0]	N/a	N/a	34.9 (15/43) [21.0; 50.9]
	MODS	51.7 (30/58) [38.2 ; 65.0]	93.3 (14/15) [68.0; 99.8]	96.8 (30/31) [83.3; 99.9]	33.3 (14/42) [19.6; 49.5]
	Xpert	50.0 (29/58) [36.6 ; 63.4])	100.0 (15/15) [78.2; 100.0])	100.0 (29/29) [88.0; 100]	34.1 (15/44) [20.5; 49.9]
	**Per sample analysis (n=178)**				
	Smear	30.0 (42/140) [20.1 ; 39.9]	N/a	N/a	27.9 (38/136) [17.1; 38.8]
	MGIT	51.4 (72/140) [40.1; 62.7]	N/a	N/a	35.8 (38/106) [22.8; 48.9]
	MODS	47.1 (66/140) [36.5; 57.7]	97.4 (37/38) [86.0; 99.7]	98.5 (66/67) [91.6; 100.0]	33.3 (37/111) [21.0; 45.7]
	Xpert	44.3 (62/140) [33.8; 54.8]	100 (38/38) [89.4; 100.0]	100 (62/62) [93.3; 100.0]	32.8 (38/116) [20.6; 44.9]

N/a=not appropriate (as 100% by definition of the gold standard).

The specificity of Xpert in all analyses was 100%. For MODS culture, the specificity was 93.3% for the per patient analysis of all TB cases due to a cross-contamination event identified by spoligotyping. Details of the contamination analysis for MODS have been previously reported [11].

#### Per sample analysis (n=178)

The sensitivity (%) of Xpert for detection of confirmed TB was 79.5 [95% CI 70.6; 88.4] while it was 84.6 [95% CI 78.1; 91.2] for MODS. The difference was not statistically significant (P=0.43). MGIT culture was significantly (p=0.03) more sensitive than Xpert with a sensitivity of 92.3% [95% CI 87.4; 97.2]. Xpert was significantly (p=0.01) more sensitive than smear which had a sensitivity of 53.8% [95% CI 39.8; 67.9].

For all TB cases (confirmed + probable), the sensitivity of 47.1% for MODS was also marginally higher than the sensitivity of 44.3% for Xpert, but the difference was not statistically significant (P=0.74). For all TB cases, the sensitivity of MGIT was 51.4% which was not significantly higher than Xpert (P=0.43). The difference in sensitivities between Xpert and smear (sensitivity 30.0%) did not reach statistical significance for all TB cases (P=0.09) (Table 2).

Xpert detected 23 positive samples which were not detected by smear, while MODS detected 27. This difference in detection of smear-negative TB was not statistically significant (p=0.24).

Xpert was negative for *M.tuberculosis* in 7% (3/42) of smear-positive samples, all of which were ‘scanty’ by smear. Among them, one pleural fluid sample was also reported as negative by both culture methods. MODS also did not detect *M.tuberculosis* in 7% (3/42) of smear positive samples all of which were ‘scanty’. Two of them were positive by Xpert (category very low and high).

### Incremental sensitivity of MODS and Xpert using multiple sputum samples from individual patients

We investigated the cumulative sensitivity of Xpert on additional samples collected from all 24 TB cases (confirmed+probable) who provided 3 sputum samples. Xpert detected *M.tuberculosis* in 50% (12/24) of first sputum samples. Testing a second sample by Xpert detected an additional two cases [+8.3%]. The sensitivity did not increase with a third sputum sample. MODS detected 62.5% (15/24) patients with the first sputum sample but did not detect any additional cases with a second or third sample.

### Time to detection

Xpert requires 2.5 hours to return a positive result. To reach a comparable sensitivity to the 79.5% of Xpert for microbiologically confirmed TB, MGIT and MODS both required around 20 days. Although MGIT culture was the most sensitive test, it took 32 days for the last culture to turn positive and reach the per-sample sensitivity of 92.3%. In positive samples, the median time to detection of MGIT and MODS was 8 days (IQR 7–13) and 13 days (IQR 9–18), respectively.

### Correlation of Ct value and smear grade

**Figure 2:**
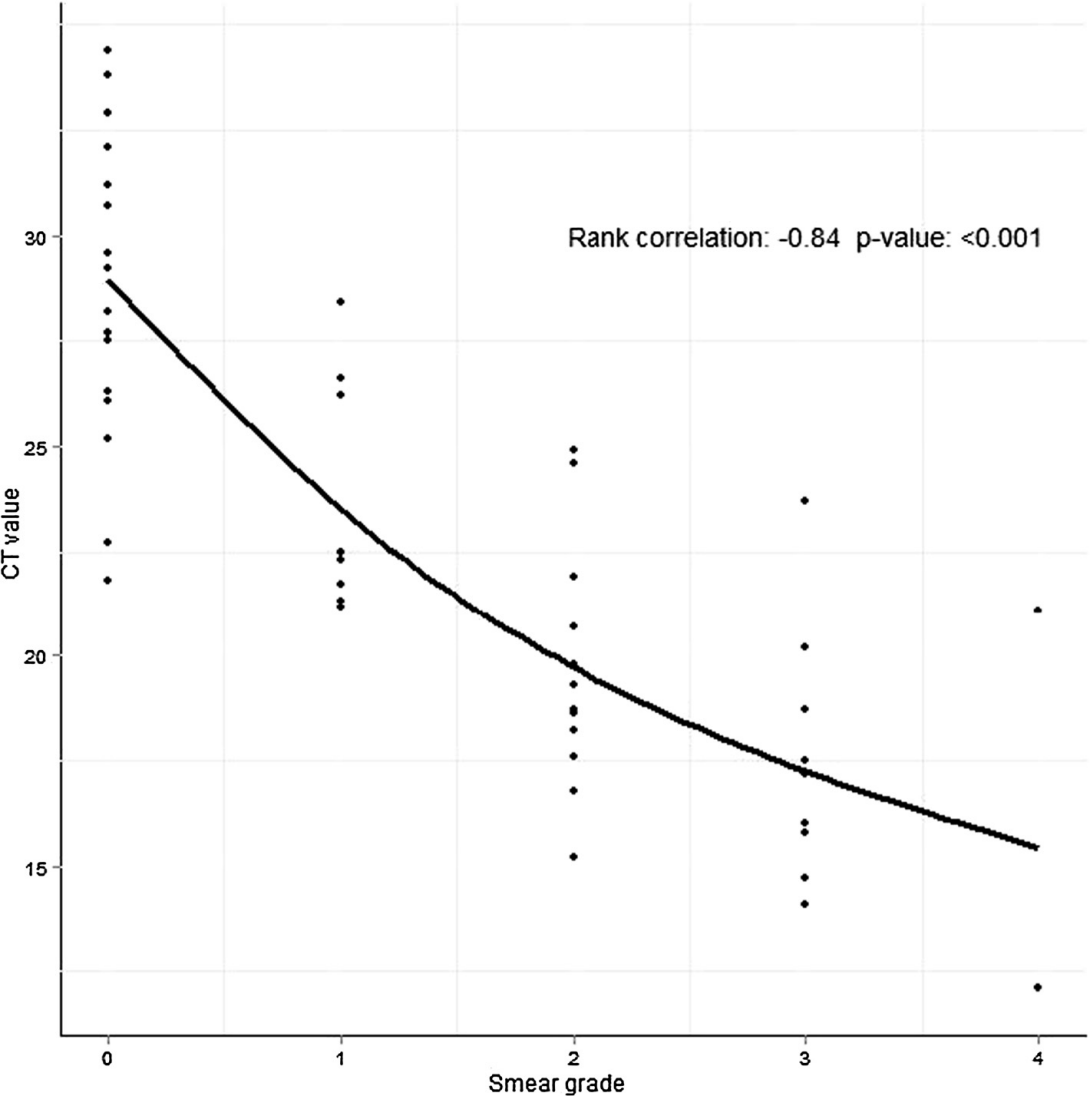
**Correlation of Ct values for Xpert probe A with Ziehl-Neelsen smear grade.** Probe A is the earliest probe in real time MTB/RIF reaction.

To investigate the correlation between quantification of bacterial load by conventional smear grading and Xpert MTB/RIF categorisation, we stratified the Ct value of probe A according to smear grade (Figure 2).

We found a strong negative association between Ct values and smear grade with higher Ct values in samples with low bacterial load (Spearman rank correlation −0.84, p<0.001). The majority of positive samples had an Xpert result category of medium (35.5%, 22/62), only 11.3% were high. Low or very low categories accounted for 25.8% and 27.4%, respectively.

### Sensitivity by sample type

Per sample sensitivity in sputum samples (n=123) based on clinical diagnosis: Sputum samples were collected from 48 patients, among whom 14 patients had two Xpert tests and 32 had three tests. The sensitivity of Xpert was 53.0% (53/100), [95% CI 40.2; 65.8], and marginally higher for MODS at 57% (57/100), [95% CI 44.3; 69.6%] among all TB cases (confirmed+ probable) (P=0.70).

Per sample sensitivity in gastric fluid samples (n=47) based on clinical diagnosis: Twenty patients had gastric fluid samples collected for TB diagnosis, among whom 12 submitted three samples and five patients submitted two samples. For gastric fluid samples, the sensitivity of Xpert was equal to MODS and 9/33 (27.3%, [95% CI 9.2; 45.4]) for both tests.

Sensitivity by sample type for confirmed TB cases: Xpert showed the same sensitivity in both sample types in TB cases with microbiological confirmation, 80.3% (n=55/66) in sputum and 81.8% (n=9/11) in gastric fluid (per sample analysis).

### Detection of Rifampicin resistance

Two patients were detected as infected with RIF resistant strains by Xpert. The test was consistently positive for RIF resistance for consecutive samples collected from the same patient.

MTBDR-*Plus* testing confirmed both strains as resistant to RIF (mutations at *rpoB* 526 and 531). Both strains were also resistant to both RIF and INH, and therefore MDR TB by MGIT SIRE. One of the two MDR strains was classified as INH susceptible by MTBDR*-Plus*. Drug resistance was not determined by MODS assay.

MTBDR-*Plus* was also performed on DNA extracted from 30 isolates from samples which were positive with *M. tuberculosis*/RIF susceptible by Xpert (one sample per patient). All were susceptible to RIF by MTBDR-Plus. There were therefore no discrepencies detected between Xpert RIF resistance detection and other tests. There was no sample with an ‘indeterminate’ result for RIF susceptibility by Xpert.

## Discussion

Xpert MTB/RIF detected 50% of children with clinically diagnosed pediatric TB, a 12%-increase over smear. Testing of multiple samples did not increase case detection for MODS and testing of a second sputum sample by Xpert detected only an additional two cases. The liquid culture techniques MODS and MGIT did not have a significantly higher sensitivity and Xpert is a ‘same-day’ test while liquid culture requires at least one week to return positive results. For maximal detection of pediatric TB, the optimal approach appears to be pairing Xpert with liquid culture (MGIT where available or MODS as an alternative). Although culture only detected a single additional case in this study, the ‘per sample’ sensitivity of MGIT culture was 14% higher overall, and culture is required to enable full drug susceptibility testing. In addition, Xpert MTB/RIF is able to rapidly detect RIF resistance. However, in this study only two patients were infected with RIF resistant strains and no robust conclusions can be drawn about the accuracy of Xpert for detection of MDR in these paucibacillary samples. The implementation of new diagnostic tests is urgently required to improve case confirmation for pediatric TB and to reduce unnecessary treatment.

Sensitivity of Xpert for detection of pediatric TB was higher in this study than two previous studies from South Africa (20.3%) and Tanzania (33.3%) [16,22]. The higher proportion of smear positive TB in our study suggests there are important differences in the patient populations between these studies, leading to the different reported Xpert sensitivities. This is likely to be due to two principal factors. Firstly, the median age of children is higher in the present study (median 8.8 years this study, 6 years Tanzania, 19 months South Africa) and confirmation of pediatric TB is generally higher in older children. For example in this study, per sample sensitivity of smear, MODS and xpert was 7.7%, 23.1% and 23.1%, respectively for children aged less than four, and 48.8%, 62.8% and 60.5% respectively, for those aged four and above. Secondly, the present study was conducted in a tertiary referral hospital for TB rather than a general hospital, therefore a higher proportion of the children were microbiologically confirmed by all methods, probably due to more advanced disease and higher bacillary loads in the samples. All studies show that Xpert increases sensitivity detection of pediatric TB over smear techniques.

Our analysis of children with 3 sputum samples suggests that the incremental value of testing multiple sputum samples from children by Xpert is moderate, with an observed 8% increase (from 50% to 58%) in sensitivity from a second sample and none from a third. Given the relatively high costs of Xpert testing, it is likely that repeat testing is only beneficial when the index of suspicion is high in this population. Further research will be needed to determine the most cost-effective algorithmic approaches to Xpert testing in children. The optimal approach will vary by setting, depending upon available resources and the patient population tested, including the ages of tested children.

Gastric fluid is often sampled in children unable to produce sputum. In samples from children with microbiologically-confirmed TB Xpert performed well on gastric aspirates, with a similar relative sensitivity to liquid culture as for sputum samples (81.8% for gastric fluid vs. 80.3% for sputum). The higher sensitivity of both MODS and Xpert on sputum for clinically confirmed cases is likely due to confounding by age: gastric aspirates were collected from younger children less likely to yield bacilli. However, relatively few gastric aspirates were available for analysis in this study (n=20), and more extensive evaluations of optimal sampling strategies in children, including string test and nasopharageal aspiration techniques, are required to maximize diagnostic efficacy [23].

Test failure (invalid and error reports) has important cost implications. The Xpert failure rate was acceptably low in this study (2.7%) and comparable with reports from demonstration sites [24].

Although MDR TB in children remains relatively rare, the consequences of delayed diagnosis are grave. The ability to rapidly detect RIF resistance in this vulnerable population is a major advance, but care must be taken, in light of the low positive predictive value in populations with a low MDR prevalence [25,26], to obtain confirmatory testing. The small number (n=2) of RIF resistant cases detected in this study does not allow robust conclusions to be drawn.

This study was performed on retrospective stored samples and not all the original samples were available for analysis which may have introduced sampling bias. It is possible that sample storage has reduced the sensitivity of Xpert in comparison with the MODS assay, which was performed on fresh samples. However, the results agree with previous reports that Xpert is a excellent test for diagnosis of pediatric TB and can be applied to both sputum and gastric aspirate samples. Xpert should be evaluated more widely using alternative sampling strategies for diagnosis of pediatric TB.

## Conclusions

In conclusion, this study shows that Xpert MTB/RIF is an accurate test for the diagnosis of pediatric TB and should be applied where available to this vulnerable population.

## Competing interests

All authors declare that they have no competing interests.

## Authors’ contributions

NTQN, DTMH, NTNL, NHD, MC designed, conceived and conducted the experiments. NDA, MW carried out the statistical analysis. DDAT, TVQ NTBT, DTMH, NTQN carried out experiments. TND, NTQ conducted recruitment, data acquisition and care of patients. NTQN, DTMH, MC, JF, MW, NDA wrote the manuscript. All authors read and approved the final manuscript.

## Pre-publication history

The pre-publication history for this paper can be accessed here:

http://www.biomedcentral.com/1471-2334/13/31/prepub

## References

[B1] Nelson LJ Wells CD Global epidemiology of childhood tuberculosis Int J Tuberc Lung Dis 2004 8 5 636 647 15137548

[B2] Corbett EL Watt CJ Walker N Maher D Williams BG Raviglione MC Dye C The growing burden of tuberculosis: global trends and interactions with the HIV epidemic Arch Intern Med 2003 163 9 1009 1021 10.1001/archinte.163.9.1009 12742798

[B3] Zar HJ Hanslo D Apolles P Swingler G Hussey G Induced sputum versus gastric lavage for microbiological confirmation of pulmonary tuberculosis in infants and young children: a prospective study Lancet 2005 365 9454 130 134 10.1016/S0140-6736(05)17702-2 15639294

[B4] Connell TG Zar HJ Nicol MP Advances in the diagnosis of pulmonary tuberculosis in HIV-infected and HIV-uninfected children J Infect Dis 2011 204 Suppl 4 S1151 1158 10.1093/infdis/jir413 21996697 PMC3192545

[B5] Marais BJ Gie RP Schaaf HS Beyers N Donald PR Starke JR Childhood pulmonary tuberculosis: old wisdom and new challenges Am J Respir Crit Care Med 2006 173 10 1078 1090 10.1164/rccm.200511-1809SO 16484674

[B6] Sandgren A Cuevas LE Dara M Gie RP Grzemska M Hawkridge A Hesseling AC Kampmann B Lienhardt C Manissero D Childhood tuberculosis: progress requires advocacy strategy now Eur Respir J 2012 40 2 294 297 10.1183/09031936.00187711 22337859 PMC3409406

[B7] Marais BJ Gie RP Hesseling AC Schaaf HS Lombard C Enarson DA Beyers N A refined symptom-based approach to diagnose pulmonary tuberculosis in children Pediatrics 2006 118 5 e1350 1359 10.1542/peds.2006-0519 17079536

[B8] Graham SM Ahmed T Amanullah F Browning R Cardenas V Casenghi M Cuevas LE Gale M Gie RP Grzemska M Evaluation of tuberculosis diagnostics in children: 1. Proposed clinical case definitions for classification of intrathoracic tuberculosis disease. Consensus from an expert panel J Infect Dis 2012 205 suppl 2 S199 208 10.1093/infdis/jis008 22448023 PMC3334506

[B9] Palomino JC Current developments and future perspectives for TB diagnostics Future Microbiol 2012 7 1 59 71 10.2217/fmb.11.133 22191447

[B10] Moore DA Evans CA Gilman RH Caviedes L Coronel J Vivar A Sanchez E Pinedo Y Saravia JC Salazar C Microscopic-observation drug-susceptibility assay for the diagnosis of TB N Engl J Med 2006 355 15 1539 1550 10.1056/NEJMoa055524 17035648 PMC1780278

[B11] Ha DT Lan NT Wolbers M Duong TN Quang ND Thi Van Thinh T Thi Hong Ngoc L Thi Ngoc Anh N Van Quyet T Thi Bich Tuyen N Microscopic observation drug susceptibility assay (MODS) for early diagnosis of tuberculosis in children PLoS One 2009 4 12 e8341 10.1371/journal.pone.0008341 20020056 PMC2791864

[B12] Oberhelman RA Soto-Castellares G Caviedes L Castillo ME Kissinger P Moore DA Evans C Gilman RH Improved recovery of Mycobacterium tuberculosis from children using the microscopic observation drug susceptibility method Pediatrics 2006 118 1 e100 106 10.1542/peds.2005-2623 16751616 PMC7617044

[B13] Blakemore R Story E Helb D Kop J Banada P Owens MR Chakravorty S Jones M Alland D Evaluation of the analytical performance of the Xpert MTB/RIF assay J Clin Microbiol 2010 48 7 2495 2501 10.1128/JCM.00128-10 20504986 PMC2897495

[B14] Chang K Lu W Wang J Zhang K Jia S Li F Deng S Chen M Rapid and effective diagnosis of tuberculosis and rifampicin resistance with Xpert MTB/RIF assay: A meta-analysis J Infect 2012 64 6 580 588 10.1016/j.jinf.2012.02.012 22381459

[B15] The Stop TB department WHO Roadmap for rolling out Xpert MTB/RIFfor rapid diagnosis of TB and MDR-TB 2010 available at http://www.who.int/tb/laboratory/roadmap_xpert_mtb-rif.pdf

[B16] Nicol MP Workman L Isaacs W Munro J Black F Eley B Boehme CC Zemanay W Zar HJ Accuracy of the Xpert MTB/RIF test for the diagnosis of pulmonary tuberculosis in children admitted to hospital in Cape Town, South Africa: a descriptive study Lancet Infect Dis 2011 11 11 819 824 10.1016/S1473-3099(11)70167-0 21764384 PMC4202386

[B17] World Health Organisation Global tuberculosis control 2011 Geneva, Switzerland: World health Organisation WHO/HTM/TB/2011.16

[B18] Population Division of the Department of Economic and Social Affairs of the United Nations Secretariat World Population Prospects The 2010 Revision. Accessed 28 May 2012. http://esa.un.org/unpd/wpp/index.htm

[B19] World Health Organisation Global Tuberculosis Programme Laboratory service in tuberculosis control: part II. Microscopy WHO/TB/98258 1998 Geneva, Switzerland: World Health Organisation

[B20] Becton Dickinson BACTEC™ MGIT™ 960 SIRE kits for the antimycobacterial susceptibility testing of mycobacterium tuberculosis. Package insert Revision: 2010/10 Accessed 28 May 2012. http://www.bd.com/ds/productCenter/245123.asp

[B21] Agresti A Caffo B Simple and effective confidence intervals for proportions and difference of proportions result from adding two successes and two failures Am Stat 2000 54 4 280 288

[B22] Rachow A Clowes P Saathoff E Mtafya B Michael E Ntinginya EN Kowour D Rojas-Ponce G Kroidl A Maboko L Increased and expedited case detection by xpert MTB/RIF assay in childhood tuberculosis: a prospective cohort study Clin Infect Dis 2012 54 10 1388 1396 10.1093/cid/cis190 22474220

[B23] Swaminathan S Rekha B Pediatric tuberculosis: global overview and challenges Clin Infect Dis 2010 50 Suppl 3 S184 194 20397947 10.1086/651490

[B24] Boehme CC Nicol MP Nabeta P Michael JS Gotuzzo E Tahirli R Gler MT Blakemore R Worodria W Gray C Feasibility, diagnostic accuracy, and effectiveness of decentralised use of the Xpert MTB/RIF test for diagnosis of tuberculosis and multidrug resistance: a multicentre implementation study Lancet 2011 377 9776 1495 1505 10.1016/S0140-6736(11)60438-8 21507477 PMC3085933

[B25] Lawn SD Brooks SV Kranzer K Nicol MP Whitelaw A Vogt M Bekker LG Wood R Screening for HIV-associated tuberculosis and rifampicin resistance before antiretroviral therapy using the Xpert MTB/RIF assay: a prospective study PLoS Med 2011 8 7 e1001067 10.1371/journal.pmed.1001067 21818180 PMC3144215

[B26] Lawn SD Nicol MP Xpert(R) MTB/RIF assay: development, evaluation and implementation of a new rapid molecular diagnostic for tuberculosis and rifampicin resistance Future Microbiol 2011 6 9 1067 1082 10.2217/fmb.11.84 21958145 PMC3252681

